# Trans-esophageal echocardiography guided closure of ventricular septal defect with 2 occluders from different incisions simultaneously: A case report

**DOI:** 10.1097/MD.0000000000023854

**Published:** 2021-05-14

**Authors:** Hui Yang, Jie Mu, Yuyi Zhao, Zizhu Chen, Haibo Song, Jin Liu

**Affiliations:** a Department of Anesthesiology, West China Hospital, Sichuan University, Chengdu; b Ultrasonic Department, Guangji Hospital, Hezhou, China.

**Keywords:** transthoracic closure, ventricular septal defects, 2 occluders, trans-esophageal echocardiography

## Abstract

**Introduction:**

Ventricular septal defect (VSD) accounts for up to 40% of all congenital cardiac malformations. Transthoracic closure of VSDs has been well described in literature. In the current report, we described a procedure to successfully close a VSD with 2 occluders from different incisions simultaneously under the guidance of trans-esophageal echocardiography (TEE), to save the patient from undergoing another surgery.

**Patient concerns:**

A 52-year-old man was referred to our clinic for repeating palpitations for 6 months without chest pain and polypnea after activity.

**Diagnosis:**

The diagnosis of VSD was established due to the findings of a juxtatricuspid VSD with a left-to-right shunt at ventricular level and mild mitral regurgitation by TTE.

**Interventions:**

A transcatheter VSD closure was firstly performed but failed to repair the VSD. After the failure of transcatheter VSD closure, the patient received transthoracic closure of VSD operated by a cardiac surgeon. The VSD was closed with 2 occluders from different incisions (median thoracic skin incision and subxiphoid incision) simultaneously under the TEE guidance.

**Outcomes:**

The patient was extubated in intensive care unit and was discharged 4 days after the operation. During the follow up, there were no significant clinical nor laboratory side-effects of the procedure found as compared to the patient's condition before the procedure.

**Conclusion:**

VSD can be closed with 2 occluders from different incisions simultaneously under the TEE guidance to save the patient from undergoing repeated surgeries. Meanwhile, TEE plays a significant role in cardiac surgery.

## 1 Introduction

Ventricular septal defects (VSDs) is 1 of the most common congenital heart defects, accounting for up to 40% of all congenital cardiac anomalies. Since many VSDs close eventually and spontaneously along with time, many VSD patients do not show symptoms until their older age.^[1]^ VSDs in adult patients have become increasingly common, consequently so does the closure procedure of VSDs.

Percutaneous closure of VSDs has been well described in literature. In the current report, treatment of a rare case of VSD was described to highlight and emphasize that VSD can be closed with 2 occluders simultaneously from different incisions under the guidance of trans-esophageal echocardiography (TEE). This procedure saved the patient from undergoing conventional surgical repair of his VSD.

## 2 Ethical statement and consent

The patient has signed the informed consent for the publication of his case. Because case reports and studies intended for quality improvement are often considered not research and do not need institutional review board approval,^[2]^ we have not gotten ethical approval form the Ethical Committee of West China Hospital, Sichuan University.

## 3 Case report

**Figure 1:**
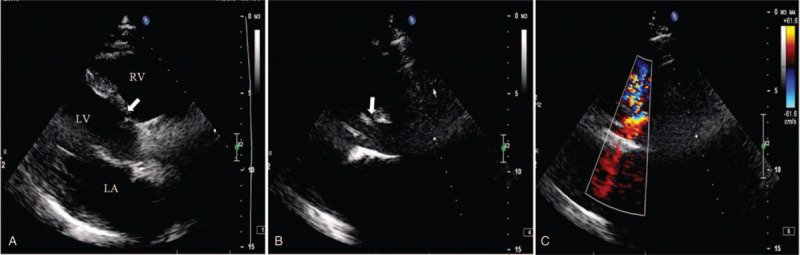
Two-dimensional Trans-thoracic parasternal long-axis view showing the ventricular septal defect (VSD) shunt from left to right. (LA = left atrium; LV = left ventricle; RA = right atrium; RV = right ventricle).

A 52-year-old man was referred to our clinic for repeating palpitations for 6 months without chest pain or polypnea after activity. No significant, relevant finding was discovered in his medical record. The patient was then diagnosed as VSD through transthoracic echocardiography (TTE), which revealed a juxtatricuspid VSD with a left-to-right shunt at ventricular level and a mild mitral regurgitation (Fig. 1). The left side of the patient's heart was enlarged, along with the existence of a 45mmHg gradient between the 2 ventricles. An operation under general anesthesia was scheduled for the patient to repair the defect since no significant contraindications was found in the patient's medical record.

Transcatheter ventricular VSD closure was firstly performed through the right femoral vein of the patient with fluoroscopy and angiography. However, a significant residual blood shunting still remained after the placement of the occluder.

**Figure 2:**
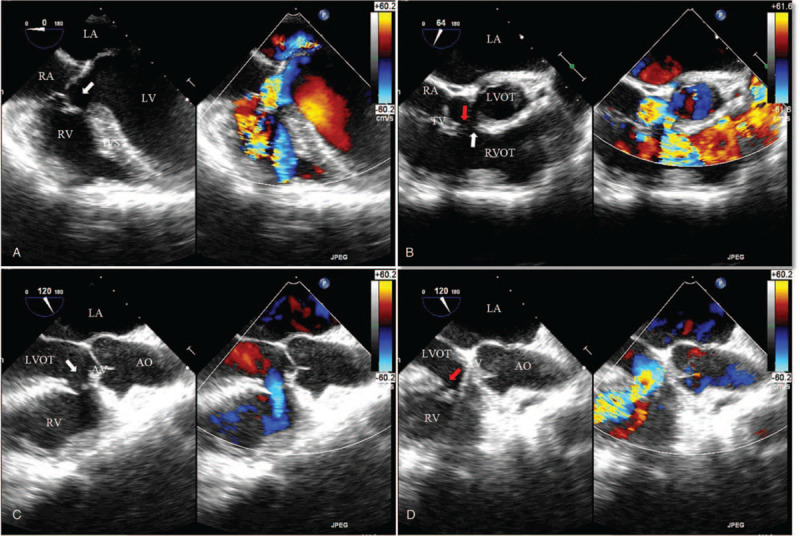
Two-dimensional and color images of the VSD (white arrow) in different views. A. TEE midesophageal (ME) Four-Chamber view; B. ME RV Inflow-Outflow view; C and D. TEE ME Aortic Valve Long Axis view. (LA = left atrium; LV = left ventricle; RA = right atrium; RV = right ventricle; AV = aortic valve; AO = aorta; LVOT = left ventricular outflow tract; RVOT = right ventricular septum; IVS = interventricular septum).

The patient was then transferred to the operation room for performing transthoracic VSD closure under general anesthesia and the guidance of TEE. Hemodynamic values remained within 30% of the preoperative values during the surgery. The supine position was used for the surgical procedure. From the midesophageal (ME) Four-Chamber view, a pre-procedure TEE illustrated a large VSD below the septal cusp of the tricuspid valve with a left-to-right shunt across the VSD (Fig. 2A, Supplemental Video 1). From the ME right ventricle (RV) Inflow-Outflow view, we found the VSD extended into the outlet portion of the left-to-right shunt and well-formed a windsock-like aneurysmal structure (Fig. 2B, the red arrow, Supplemental Video 2) that had multiple fenestrations (Fig. 2B, the white arrow, Supplemental Video 2) from the right ventricular side. The ME Aortic Valve Long Axis view showed us that there were 2 fenestrations (Fig. 2C, the white arrow and Fig. 2D, the red arrow, Supplemental Video 3) on the right ventricular side of the left-to-right shunt.

**Figure 3:**
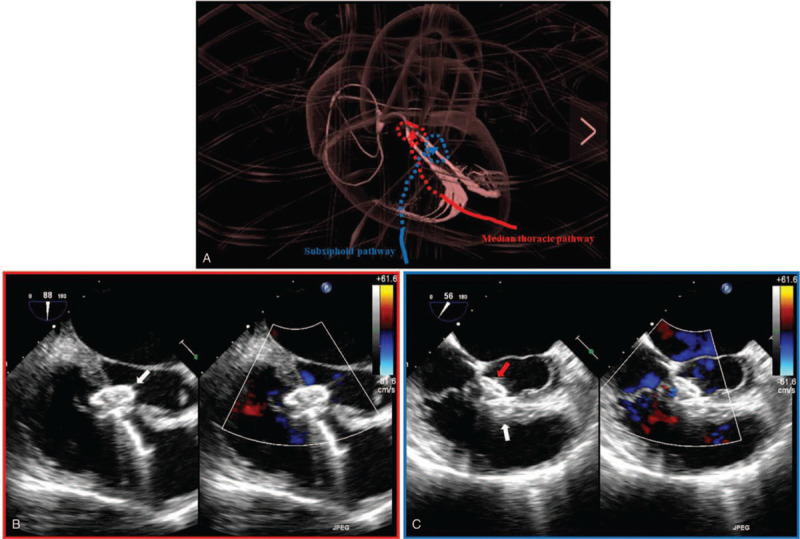
The Two pathways for 2 occluders placement. **A**. a schematic view of 2 pathways; B. TEE image showing the first occluder (white arrow) placed via the pathway through a small median thoracic incision; C. TEE image showing the second occluder (red arrow) placed via the pathway through a small subxiphoid incision. (LA = left atrium; LV = left ventricle; RA = right atrium; RV = right ventricle; LVOT = left ventricular outflow tract; IVS = interventricular septum).

**Figure 4:**
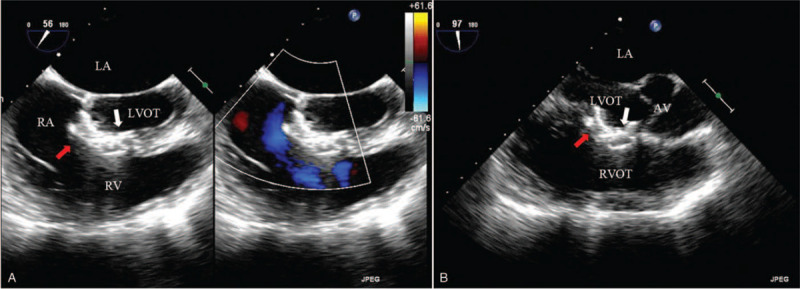
Two occluders (white and red arrows) covering VSD without residual shunt. **A**. TEE ME RV Inflow-Outflow view; **B**. TEE ME aortic valve long axis view; (LA = left atrium; LV = left ventricle; RA = right atrium; RV = right ventricle; AV = aortic valve; AO = aorta; LVOT = left ventricular outflow tract; RVOT = right ventricular septum; white arrow: the first occluder; red arrow: the second occluder).

The intervention was done as previously described for VSD closure using different devices ^[3]^ (Fig. 3A). Briefly, antibiotics and heparin (1 mg/kg) were administrated intravenously before the operation. A median thoracic skin incision between 1 to 2 centimeters was made. The subcutaneous tissue was dissected to the fourth left parasternal intercostal space. The intercostal muscles were dissected to establish the surgical route. The right ventricle free wall was exposed and punctured with a trocar. A 0.035-inch guide wire was placed in the trocar. After the guide wire was passed through the VSD, the trocar was removed and a dilator and a 7 Fr delivery sheath were advanced through the VSD to the left ventricle along the guide wire. After removing the guide wire, the dilator and the sheath, the first selected occluder (Beijing Huayi Shengjie Co, Ltd, Beijing, China), which had a 12-mililiter(mm) diameter of symmetric double-disk and a 3-mm waist height, was deployed through the delivery sheath (Fig. 3B, Supplemental Video 4). All the procedure was performed under the TEE guidance. The TEE was also used to reevaluate the shape and position of the placed occluder, the presence of a residual shunt, and valvular regurgitation before and after occluder release. The same procedure was performed to repair the second fenestration with the exceptions that the access to the surgery site was through a small subxiphoid incision and the diameter of the deployed symmetric double-disk was 14-mm and the height of the waist was 4-mm (Fig. 3B, Supplemental Video 5). Similarly, TEE was used to reassess the shape and position of the placed occluder, the presence of a residual shunt, and valvular regurgitation before and after occluder release. Post-procedure TEE showed that, being adjacent to each other, the 2 occluders covered the vast majority of the VSD with a trace shunt remaining (Fig. 4, Supplemental Video 6).

Finally, the delivery sheath was withdrawn and the patient was sent to the intensive care unit. The whole process lasted less than 60 minutes. The patient was extubated in intensive care unit and discharged 4 days after the operation. During the 1.5-year follow up, no significant clinical nor laboratory findings suggesting hemolysis were found. Furthermore, electrocardiogram (ECG) showed normal sinus rhythm with no heart block. Echocardiography showed that the devices were in position with a mild tricuspid regurgitation (TR), which have existed before the procedure.

## 4 Discussion

Ventricular septal defect (VSD) is 1 of the most common congenital heart defects. Both surgical and percutaneous device closure of VSDs have drawbacks and limitations. In 2007, Xing and his colleagues reported 11 successful cases of perimembranous VSD closures by minimally invasive transthoracic device closure technique using a newly designed delivery system under the guidance of TEE. Since then, minimally invasive transthoracic device closure of VSDs under the guidance of TEE has been increasingly and successfully performed with excellent preliminary and midterm outcome in China and some European countries. The most prominent feature of this new system is its short dimension. By gently pressing the free wall of the right ventricle, the best point for puncture closest to the perimembranous ventricular septal defect can be ascertained. It is also possible to adjust the sheath to be parallel to the defect, which eliminates some defects of transcatheter VSD closure system. In our case, the patient's VSD was successfully closed through 2 small incisions with minimal injury.

In addition, there is no doubt about the importance of TEE in interventional cardiology. Traditionally, cardiovascular procedures in the catheterization laboratory are guided by fluoroscopy and angiography. Advances in echocardiography overcome most limitations of conventional imaging techniques. For instance, echocardiography is portable, offers real-time imaging, provides accurate anatomic and physiologic assessment of the target structure, and facilitates appropriate patient selection. It is conducive for early identification of complications and the patient follow-up checking after the procedure. All of these features of echocardiography increase the likelihood of a successful post-procedural outcome.^[4]^ Given the specific location of the patient's VSD in the current case report, TEE had more advantages than preoperative TTE for diagnosis and guidance of the intervention. TEE helped us diagnose the VSD with 2 fenestrations from the right ventricular side, which laid solid foundation for the successful VSD closure. Intraoperative TEE has been recognized as a monitoring approach that has a major impact on the outcome of the repair of congenital heart disease.^[5]^

The novelty in our case was that we deployed 2 occluders through 2 different incisions at the same time to save the patient from another operation. Compared to the conventional surgical closure and other transcatheter device closure techniques for VSDs, the obvious advantages of our procedure include less surgical trauma and no radiation. The entire procedure can be done without the support of cardiopulmonary bypass (CPB) and blood transfusion. Moreover, cardiac surgeons are more familiar with the intracardiac anatomy and know how to avoid damage to myocardial and adjacent tissues during the procedure because they are doing intracardiac operations almost every day.

From different angles of the TEE views, we found that the VSD had 2 fenestrations from the right ventricular side but only 1 from the left side. Although the 2 fenestrations from the right side were adjacent to each other, they extend in different directions. Accordingly, we closed the VSD from different directions through different incisions. That was also the reason why the VSD was unable to be closed through transcatheter. Without limitations related to the age and body weight of patients, the application of our technique mainly depends on the location of VSD and operating pathway, which could not be done beyond the extensive experience of cardiac surgeons and echocardiologists. If the VSD fenestrations lean back, this technique may not be suitable for the patients.

To the best of our knowledge, the operational procedure reported here is the first of its kind. We assume that by using TEE, the procedure can be performed in a diverse range of situations. It is not only easier, but also safer.

## Author contributions

Manuscript preparation was by Hui Yang; manuscript review and correction were by Jin Liu; videos were prepared by Jie Mu, Yuyi Zhao and Zizhu Chen.

Conceptualization: Hui Yang, Jin Liu.

Software: Jie Mu and Yuyi Zhao.

**Conceptualization:** Haibo Song.

**Data curation:** Zizhu Chen.

**Methodology:** Jie Mu.

**Writing – original draft:** Hui Yang.

**Writing – review and editing:** Yuyi Zhao, Jin Liu.

## Supplementary Material

Supplemental Digital Content

## Supplementary Material

Supplemental Digital Content

## Supplementary Material

Supplemental Digital Content

## Supplementary Material

Supplemental Digital Content

## Supplementary Material

Supplemental Digital Content

## Supplementary Material

Supplemental Digital Content

## References

[1] Penny DJ Vick GW. Ventricular septal defect. Lancet 2011;377:1103–12. 21349577 10.1016/S0140-6736(10)61339-6

[2] Akhondzadeh S. Do case reports require ethical approvals? Case Rep Clin Pract 2016;1:67.

[3] Ou-Yang WB Li SJ Wang SZ. Echocardiographic guided closure of perimembranous ventricular septal defects. Ann Thorac Surg 2015;100:1398–402. 26234660 10.1016/j.athoracsur.2015.05.036

[4] Patrianakos AP Zacharaki AA Skalidis EI. The growing role of echocardiography in interventional cardiology: The present and the future. Hellenic J Cardiol 2017;58:17–31. 28163148 10.1016/j.hjc.2017.01.008

[5] Patel JK Glatz AC Ghosh RM. Accuracy of transesophageal echocardiography in the identification of postoperative intramural ventricular septal defects. J Thorac Cardiovasc Surg 2016;152:688–95. 27183884 10.1016/j.jtcvs.2016.04.026 PMC7178077

